# Towards patient-specific cardiovascular modeling system using the immersed boundary technique

**DOI:** 10.1186/1475-925X-10-52

**Published:** 2011-06-17

**Authors:** Wee-Beng Tay, Yu-Heng Tseng, Liang-Yu Lin, Wen-Yih Tseng

**Affiliations:** 1 High Performance Computing & Environmental Fluid Dynamic Laboratory, Department of Atmospheric Sciences, National Taiwan University, Taipei, Taiwan; 2 National Taiwan University Hospital, Taipei, Taiwan; 3 Center for Optoelectronic Biomedicine, National Taiwan University College of Medicine, Taipei, Taiwan

## Abstract

**Background:**

Previous research shows that the flow dynamics in the left ventricle (LV) reveal important information about cardiac health. This information can be used in early diagnosis of patients with potential heart problems. The current study introduces a patient-specific cardiovascular-modelling system (CMS) which simulates the flow dynamics in the LV to facilitate physicians in early diagnosis of patients before heart failure.

**Methods:**

The proposed system will identify possible disease conditions and facilitates early diagnosis through hybrid computational fluid dynamics (CFD) simulation and time-resolved magnetic resonance imaging (4-D MRI). The simulation is based on the 3-D heart model, which can simultaneously compute fluid and elastic boundary motions using the immersed boundary method. At this preliminary stage, the 4-D MRI is used to provide an appropriate comparison. This allows flexible investigation of the flow features in the ventricles and their responses.

**Results:**

The results simulate various flow rates and kinetic energy in the diastole and systole phases, demonstrating the feasibility of capturing some of the important characteristics of the heart during different phases. However, some discrepancies exist in the pulmonary vein and aorta flow rate between the numerical and experimental data. Further studies are essential to investigate and solve the remaining problems before using the data in clinical diagnostics.

**Conclusions:**

The results show that by using a simple reservoir pressure boundary condition (RPBC), we are able to capture some essential variations found in the clinical data. Our approach establishes a first-step framework of a practical patient-specific CMS, which comprises a 3-D CFD model (without involving actual hemodynamic data yet) to simulate the heart and the 4-D PC-MRI system. At this stage, the 4-D PC-MRI system is used for verification purpose rather than input. This brings us closer to our goal of developing a practical patient-specific CMS, which will be pursued next. We anticipate that in the future, this hybrid system can potentially identify possible disease conditions in LV through comprehensive analysis and facilitates physicians in early diagnosis of probable cardiac problems.

## Background

Research commonly uses computational fluid dynamics (CFD) simulation to investigate cardiovascular problems. Most of the research in this field focuses on a specific region of the heart [1,2] rather than on the entire heart. A three-dimensional (3-D), CFD based, patient-specific cardiovascular modelling-system of the entire heart is currently underdeveloped. This study introduces a cardiovascular modelling system (CMS) which may detect possible disease conditions and facilitate early diagnosis prior to heart failure, using hybrid CFD simulation and time-resolved magnetic resonance imaging (MRI).

Emphasis on the left ventricle (LV) of the heart dates back to the 1970s [3]. Early experimental studies reported an eddy generation in the LV during ventricular filling, thought to cause early partial closure that prevents regurgitation. Recent research [4] notes that the flow dynamics found in the LV reveal important information about overall cardiac health, useful in early diagnosis of patients with potential heart problems. However, later studies [5,6] have shown that the valve closure is because of the developing adverse pressure gradient which causes the flow to decelerate well before it reverses. Mcqueen and Peskin [7] conducted computational studies to investigate a natural or prosthetic mitral valve as early as 1982, and later developed a successful 3-D heart simulation based on idealized hemodynamic conditions [8]. They have used the immersed boundary method (IBM) to accommodate complex geometries, moving wall, and fluid-tissue interaction. This approach is much more suitable than other numerical methods based on structured or unstructured grids because it allows for large grid deformation and is efficient. Appropriate data on flow rates and pressures of the heart at various times throughout a cycle enables visualizing and detecting peculiarities occurring within the heart during simulation, as well as detailed analysis of the flow pattern within the ventricles of the heart. However, the potential problem with the approach of Peskin is that the use of the smoothing function results in a "spreading" effect. In other words, the immersed boundary does not remain a "sharp" interface, thus reducing accuracy.

Vortex formation in the LV has also attracted recent attention. Fortini et al. [8] investigated the effect of mechanical heart valves (MHV) on the flow characteristic of the blood inside an LV model. The three types of tested MHV include (a) a one-way, hydraulic valve; (b) a monoleaflet valve; and (c) a bileaflet valve. The first configuration most resembles the natural valve, while the modelled LV is a silicone rubber conical sack, which is flexible and transparent. A comparison of vorticity shows that valve (a) produces the simplest plot with only two oppositely signed vortices. The vorticity fields generated by valves (b) and (c) are more complicated, generating four to five vortices, which are less orderly compared to the valve (a) case.

Several numerical studies of flow within LV also show the complexity of vortex formation. Domenichini et al. [9] used a mixed spectral-finite differences method to simulate the 3-D fluid dynamics inside the LV of the heart during diastole. They analyzed the sensitivities of several governing parameters, including eccentricity, the Stokes number, and the Strouhal number, and found well-defined vortice structures, regardless of these parameters. As eccentricity increases, the flow field changes smoothly from axisymmetric to complex 3-D structures when the values are similar to physiological ones. The effect of the Stokes number on the flow is rather weak. As the Strouhal number decreases, the effect of convection increases and the entry jet extends more deeply into the ventricle. Instability may follow and result in weak turbulence. However, their simplified model only comprises the LV of the heart. Hence, a more sophisticated simulation, including the entire 3-D heart, is required. Nevertheless, their analysis theoretically presents the possible flow structure associated with diverse parameters.

Saber et al. [10] used patient-specific MRI images to construct the geometry of the LV of a 3-D heart in a numerical simulation. Hence, the fluid structure interaction (FSI) prescribes, rather than influences, the geometry of the LV at various instances. The valves of LV are represented by 2-D planar models. The simulation is based on another commercial CFD solver, the Star-CD. Saber et al. [10] could not specify the inflow/outflow rate at the mitral/aortic valves as a boundary condition because of limited measurements. Therefore, a uniform, constant pressure was prescribed at the mitral and aortic valves. Their model captured the 3-D contraction and expansion phases of the LV. However, they underestimated areas of the mitral and aortic valves with overestimated velocities. This could be due to the low resolution of MRI and interpolation uncertainties. Saber et al. [10] indicated the importance of clear MRI images to construct the geometry of LV in simulation. However, blurring or ghosting artifacts are common in MRI images due to breathing motion and bowel movement [11]. Particularly for time-resolved 3-D data acquisitions, large amounts of data require measurement durations that often exceed normal human breath-holding capabilities. These poor quality images hinder accurate analysis of the heart. Markl et al. [12] used an improved navigator-gated time-resolved, phase contact MRI (PC-MRI) velocity mapping based on real-time adaptive k-space reordering, combined with a wider data acceptance window to improve image quality [12]. This system reconstructs 3-D images of the heart over a cardiac cycle, as well as time-resolved 3-D hemodynamic velocity fields, yielding excellent images with moderate blurring and minor ghosting artifacts. Current available data comprises of both healthy volunteers and patients with cardiac problems for comparison.

This study develops (and introduces) a 3-D CFD based, patient-specific cardiovascular modelling system in order to facilitate the physician's early diagnosis of possible cardiac problems in practical application. The tools comprise of a 3-D CFD model to simulate the heart and a 4-D (3-D in space and 1-D in time) PC-MRI system. This hybrid system can potentially identify possible disease conditions in the LV through analyzed vorticity, kinetic energy, hemodynamics, pressure, and shear stress. Approximately 30% of all heart attacks are fatal [13]. The availability of such a system can avoid sudden heart attacks and save more lives in advance. Similar to Saber et al. [10], this study uses CFD simulation to construct an accurate model representation of the heart flow within the cardiac cycle and the resulting vortex dynamics in the LV. However, unlike Saber et al. [10], the current construction of the heart model did not directly use MRI images to obtain the geometrical data due to the difficulty of three-dimensional motions and the lack of information about the fiber connection. We initialized the geometry and fiber characteristics based on the approach of Mcqueen and Peskin [7]. To achieve enhanced accuracy, we take advantage of the IBM code described by Mcqueen and Peskin [14] rather than a commercial software. The IBM is more suitable for large movement simulation because it does not suffer from grid quality deterioration, commonly seen in structured grids. The current study simulates the entire heart based on the anatomy and mechanical properties of heart muscle fibers, extensively documented by physiologists. The geometry of the heart changes with time according to the FSI between fluids and muscles; hence, it is more accurate to simulate the changing geometry of the heart.

As a first step, we concentrate on verifying the results of CMS with clinical data in this paper. These results and comparisons are essential for the subsequent model improvements and practical implications. The next section describes the methodology of the cardiovascular modelling system, followed by a detailed description of the 4-D PC-MRI system and its capabilities. The numerical method developed by Mcqueen and Peskin [14] will be explained briefly. Our results involved hemodynamic comparison, vortex and kinetic energy analysis which show some agreements with the compared cases. The conclusion summarizes the overall performance and the specific areas which require further investigations.

## Methods

### Patient-specific Cardiovascular Modelling System

**Figure 1:**
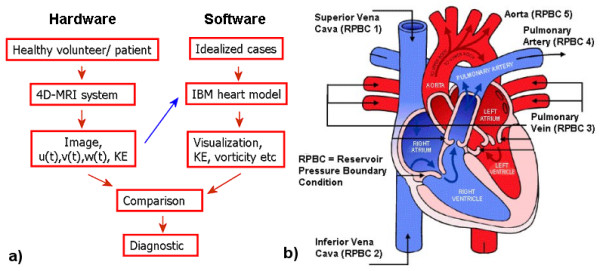
**The proposed Cardiovascular Modeling System (CMS) and sketch of heart diagram**. a) Fundamental methodology of the Computational Fluid Dynamics based, patient-specific CMS. b) Diagram of the heart with the five key boundary sources (Figure modified from Abdallah [28]).

Developing an accurate CFD based patient-specific cardiovascular modelling-system involves integrating analyzed data from the 4-D PC-MRI system (hardware) into the IBM heart model (software). Figure 1a shows the fundamental methodology. The 4-D PC-MRI system scans healthy volunteers, as well as patients with cardiac problems. The raw data comprises of images and the hemodynamic velocity of the heart over an entire cardiac cycle. The 3-D IBM heart model [14] requires initial and boundary conditions for the pressure, which can be obtained from idealized cases or documented estimations of healthy adults. This information allows us to investigate various scenarios and their effects on the LV of the heart. The quasi-realistic simulation provides very useful heart flow information for diagnostics, such as visualization, velocity field, kinetic energy (KE), vorticity, and pressure.

In the CMS, the boundary conditions are specified at five major inflow and outflow sources, including 1) superior vena cava (SVC); 2) inferior vena cava (IVC); 3) pulmonary vein (PV); 4) pulmonary artery (PA); and 5) aorta, as shown in Figure 1b. These five sources dictate how the blood flows in and out of the heart and are crucial. The SVC and IVC are two large veins in charge of transporting de-oxygenated blood to the right atrium of the heart. The SVC is formed by the left and right brachiocephalic veins and blood through the SVC enters the right atrium through the upper right front of the heart. Similarly, the blood in the IVC enters through the lower right, backside of the heart. The PV (four in reality, but simplified to only one in the 3-D heart model) carries oxygenated blood from the lungs to the left atrium (LA) of the heart and delivers de-oxygenated blood from the heart to the lungs. The aorta, the largest blood vessel in the body originating from the LV of the heart, transports oxygenated blood to all parts of the body. Previous research [9,15,16] shows that the filling dynamics of the LV contain vital health information of the heart. This study therefore concentrates on the flow dynamics of the LV and the PV, which directly affects the blood entering the LV.

The CFD heart model aids in close investigation of the entire process within the LV to examine the possibility of observing disease-related dysfunctions in the dynamics of transmitral blood flow during early LV diastole. Combining the non-invasive 4-D PC-MRI information with the quasi-realistic heart model provides a unique opportunity to evaluate cardiovascular hemodynamic before complete heart failure. Here, we emphasize more on the initial comparison between the modelled flow dynamics in LV with the clinical data for a healthy subject to obtain a better understanding of the current technique.

### 4-D PC-MRI System

The latest 4-D time-resolved PC-MRI, located at the National Taiwan University Hospital, provides enhanced and more realistic physiological information for the CFD flow simulation. This system acquired images using a 3T MR imaging system (Magnetom Trio; Siemens, Erlangen, Germany, gradient performance: 40 mT/m in 200 s) with standard body coil [17], which allows the reconstruction of 3-D images of the heart over a cardiac cycle. However, getting clear and accurate images is not straightforward due to various problems, such as insufficient respiration control, artifact generation, and limited signal-to-noise ratio [12]. Post-processing is required to identify, check, and extract relevant heart flow fields over the cardiac cycle (19 contiguous volume image frames). We obtained images at intervals of approximately 45 milliseconds. The field of view (FOV) is 307 × 230 mm^2 ^with matrix = 256 × 192 and the section thickness is 6 mm. This forms the volume images at 256 × 192 × 8 (x, y, z), which approximates to an actual size of 307 × 230 × 48 mm^3^. Each "z=" plot below represents the orthogonal coordinate section of the xy plane. Only 8 slices in the z direction are used due to the constraint of the PC-MRI system. Hence, the images can only provide qualitative flow dynamics in patients and volunteers. We anticipate the CMS shall provide us better details with a reasonable comparison with the PC-MRI system.

**Figure 2:**
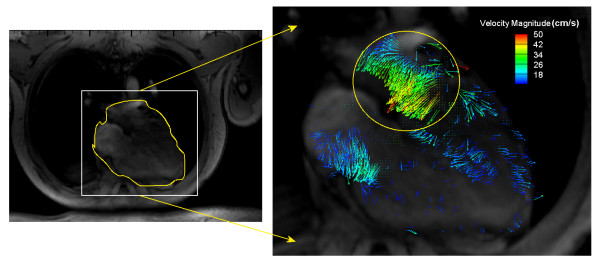
**One of the heart image slices using the 4-D Phase contrast (PC)-MRI system at 256 × 192 resolution**. The color indicates its velocity magnitude.

The test subject of the 4-D PC-MRI system for this initial test is a healthy 35 year-old female volunteer with no underlying disease. The study was approved by the Institution Review Board (IRB) at the hospital. MRI images over an entire heart cycle were taken. A snapshot image taken at T = 0.2 in Figure 2, corresponds to the diastolic phase (1T is equivalent to a whole heartbeat), and shows one of the heart image slices at a resolution of 256 × 192. The colored arrows indicate the velocity vectors of the blood flow. A high concentration of vectors in the circled region corresponds to the diastolic phase when large amounts of blood enter the LV. Note that, in the current paper, the results obtained from 4-D PC-MRI system are only used for comparison and verification purpose, however, its output can be feed in to the CMS as shown by the blue arrow in Figure 1a. The results will be shown in a subsequent paper.

### 3-D Cardiovascular Model System (CMS) and Numerical Method

The IBM algorithm is based on Lai and Peskin [18], and verified using the time periodic vortex street behind a circular cylinder. The 3-D CMS is based on the IBM model developed by Mcqueen and Peskin [14] and is more adequate for modelling elastic and contractile fibers of the heart compared to other variations of the IBM [19,20]. The governing equations are based on the 3-D incompressible Navier-Stokes equations with immersed boundary forcing given by:(1)(2)(3)(4)(5)

In this case, ***x **= (x, y, z), **u**(**x**, t) = (u(**x**, t), v(**x**, t), w(**x**, t))*, is the fluid velocity and *p(**x**, t) *is the fluid pressure. *μ *and *ρ *represent the fluid viscosity and density respectively. The force density (with respect to *d**x ***= *dxdydz*) acting on the fluid is ***f**(**x**, t) = (f_x_(**x**, t), f_y_(**x**, t), f_z_(**x**, t))*. The ***s ***tracks a material point of the immersed boundary and the boundary force density (with respect to *ds*) is ***F**(s, t) = (F_x_(s, t), F_y_(s, t), F_z_(s, t))*. Equations (3) and (4) estimate the interaction between the immersed boundary and the fluid. Equation (5) represents the boundary force resulting from the boundary configuration at time *t*, where the function ***S ***satisfies a generalized Hooke's law if the boundary is elastic.

**Figure 3:**
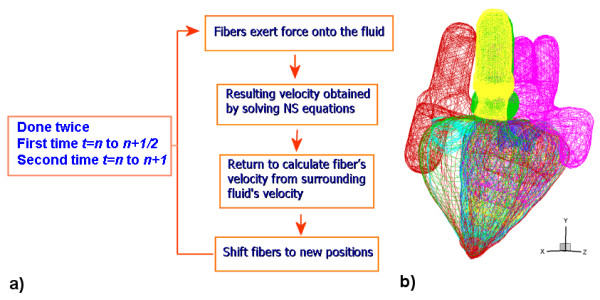
**a) The flowchart and b) computational meshes of the 3-D CMS based on the immersed boundary method**.

Two sets of grids, fixed and moving, are adopted in the CMS. The fixed grid represents the Cartesian grid *x, y, z *which covers the entire fluid domain. The moving grid represents the boundary or fibers of the heart. At each time step, the force acting at each point on the fiber is calculated using Equation (5). These fibers exert force onto the fluid, represented by the Cartesian fixed grid, and the force on the fluid is calculated using Equation (3). The resulting velocity at time step (*n+1/2*) is then obtained by solving Equations (1) and (2) using the fractional step method, which solves the momentum and the pressure Poisson equations. With this, one can return to interpolate the velocity of the fiber from its surrounding velocity of the fluid. The fibers then move to their new positions at *t = n+1/2 *based on their velocities. With the known velocities and positions of the fibers at *t = n+1/2*, the solution process is repeated, but now they are used to take a full time step from *t = n *to *n+1*. This results in a time-centered or Crank-Nicolson scheme which has "formal" second-order accuracy [18]. The whole process is sketched in Figure 3.

Constructing the 3-D CMS includes a few steps. The most fundamental component is the boundary points, which are joined together to form fibers. The fibers with the same number of points are clustered together as a group, while a bunch is made up of groups. The combined bunches form the heart. Hence, the entire heart model is made up of about 4,000 fibers consisting of about 600,000 boundary points. The fiber connections are based on studies of the hearts of dog and hogs which were bathed in a substance that dissolves the connective tissue between the muscle fibers. More details about the construction of the heart can be found in Mcqueen and Peskin [14]. Fiber elasticity is nonlinear and time dependent and possesses different values at various areas because it performs diverse functions. Local fiber strain determines the fiber tension, and this stress-strain relationship drives the contraction and relaxation of the cardiac muscle. The characteristics of fiber properties can be found in Kovacs et al. [21]. Hence, this can be defined as a fully FSI study.

**Figure 4:**
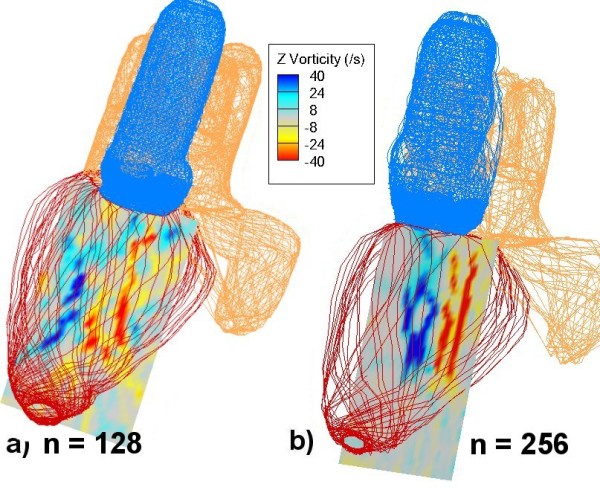
**Vorticity plot comparison at T = 0.2 for a) n = 128 and b) n = 256 grids**.

The fixed Cartesian grid uses uniform 128 × 128 × 128 grid points with a domain size of approximately 17 × 17 × 17 cm cube. The width of 17 cm is based on a domain of 64 meshwidths. A mitral ring radius of 5.85 meshwidths represents the 10 cm circumference of the human mitral ring. A grid refinement study was carried out by running the simulation partially up to T = 0.2 with a grid size of 256 × 256 × 256 grid points. Qualitative visualization shows the similarity between both heart expansion and contraction of the 128^3 ^and 256^3 ^grids at the early stage. Figure 4 shows the vorticity plots of a slice of the simulated heart for the two different grid resolutions at T = 0.2, which corresponds to the filling period. This filling period will be explained further in the later sections. The color contours are similar (a pair of vortices) but their distribution differs slightly, as expected. This could be attributed to the slight difference at which blood enters the LV.

Assuming a peak physiological Reynolds number (Re) of 6000, the current number of grid points is clearly insufficient to resolve the boundary layer in the simulation. At Re ≈ 6000, turbulent flow is expected. As a preliminary study, the current simulation assumes a laminar flow and does not use any turbulence model. Note that the main purpose of this study is not to create an exact model of the heart, which will require very high computational resources to resolve the detailed forces and FSI. This is also due to the fact that the immersed boundary method of Peskin [18] is not a sharp interface method. Therefore, it will not be able to give a sharp representation of the heart [22]. On the other hand, it is able to better represent the elastic immersed boundary. Hence our objective in this paper is to adequately capture a qualitative picture of the effects of the heart model wall and the FSI.

A new single heartbeat, together with the initial transient, requires 57,344 time steps. An initial transient period is required because the simulation may not run successfully if the LV is empty. The LV must be filled with sufficient blood before conducting the actual cardiac cycle. This filling period runs from t = 0 to 0.34 s. Therefore, our analysis of the cardiac cycle starts from t = 0.34 s to t = 1.14 s (the complete cardiac cycle T lasts 0.8 s). The time t = 0.34 s corresponds to the onset of the diastolic phase. Hereafter, the time is normalized by the complete cardiac cycle (T = 1.0) for simplicity.

Simulation of a single heartbeat takes approximately 42 hours on a Linux cluster comprising the Intel Xeon Woodcrest Quad-Core 5345 2.33GHz processors with openmp parallelization enabled. Subsequent heartbeats require 32,768 time steps. With an assumed heartbeat lasting 0.8 s and time step 2.44 × 10^-5 ^s, the current simulation obtains results from the first heartbeat after the initial transient.

As mentioned earlier, the boundary condition for the CMS is specified at five locations of the heart. Cyclic pressure boundary conditions are specified. Similar to Mcqueen and Peskin [14], this work imposed a fixed reservoir pressure boundary condition (RPBC) throughout the cardiac cycle comparable to the boundary condition of Saber et al. [10]. Table 1 shows the RPBC at various sources of the heart. The density and kinematic viscosity of the fluid are 1.0 g/cm^3 ^and 0.03125 cm^2^/s[23]. Although the solver we used here is the same as that in Kovacs et al. [21], we analyzed the results by comparing against clinical data in terms of hemodynamic, vorticity, and kinetic energy (KE).

**Table 1:** RPBC at different sources of the heart

Sources	SVC, IVC	PV	PA	Aorta
Reservoir pressure/mmHg	100	15	5	80

## Results

### Hemodynamic comparison

**Figure 5:**
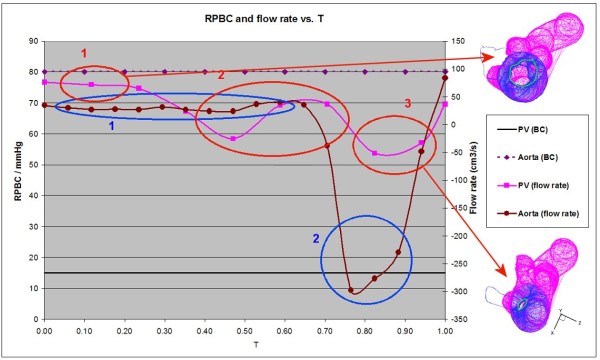
**The reservoir pressure boundary condition (RPBC) and flow rate throughout one cardiac cycle**.

Figure 5 shows the PV and aorta RPBC as well as their flow rate variation for one cardiac cycle. In the simulation, between T = 0.0 and 0.25 (initial LV filling phase also known as the E-wave [24], marked as the red circle 1 in Figure 5, the inflow rate at the PV is almost constant where the blood enters the heart. At this instant, the mitral valve in the simulation also opens, shown by the green circle at the top right hand side of Figure 5. In the middle of the LV filling phase (just after T = 0.4, red circle 2), the flow rate decreases, reverses, and then increases again (circle 2, Figure 5). This indicates that some amount of blood is flowing out of the PV, resulting from a higher LA pressure due to the filled-up blood in LA. This is not surprising because the reservoir pressure is always constant. With higher LA pressure, backflow occurs and some blood begins to flow out, resulting in the negative PV flow rate. After T = 0.5, the inflow resumes briefly, corresponding to the second filling phase, commonly known as the A-wave [24], which is due to atrial contraction. The earlier backflow lowers the pressure in the LA and resumes blood flow. However, the PV flow rate decreases again after T = 0.7. The diastole phase ends and the systole phase begins. During systole, the inflow decreases to zero and changes to outflow. A stronger outflow is again evident between T = 0.75 to 1.0 (red circle 3, Figure 5) because of higher pressure in the LA resulting from earlier inflow of blood.

**Figure 6:**
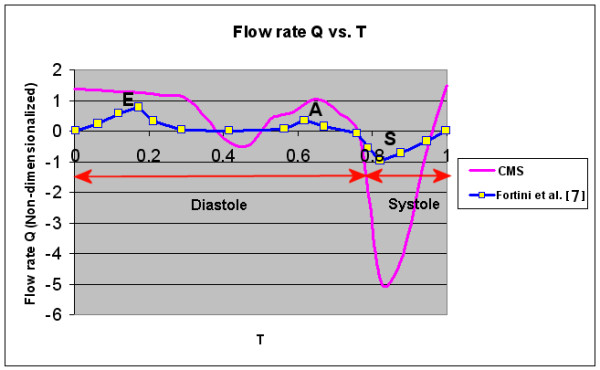
**Magnitude comparison between the inlet flow rate *Q *of the LV for CMS and Fortini et al**. [8]**throughout one cardiac cycle**. E, A and S refer to the E-wave, A-wave and S-wave, respectively.

Figure 5 shows a constant aorta RPBC 80 mmHg, with no significant difference in the flow rate of the aorta from T = 0 to 0.65. This is because this period (blue circle 1) corresponds to the initial filling of blood in the LV. The pressure in the LV is low, compared to aorta pressure, and hence only a small amount of inflow exists. After T = 0.65, the systole phase begins (blue circle 2). During this period, large amounts of oxygenated blood start to flow out through the aorta to other parts of the body. In Figure 6, the magnitude of the flow rate is non-dimensionalized using the stroke volume and the cardiac cycle T. The stroke volume refers to the difference in the volume of the LV between the end-diastolic and the end-systole phase. This flow rate is compared against the similar clinical data from Fortini et al. [8], Baccani et al. [24], and Domenichini et al. [25]. Their data are similar except that they are non-dimensionalized differently. *Q *represents the flow rate through the mitral during the diastole (0.00-0.75 T, containing the E- and A-waves), and through the aortic valve during the systole (0.75-1.00 T, containing the S-wave). The graph of Fortini et al. in Figure 6 is obtained by combining flow rate from the mitral value and aorta during the diastole and systole phase. Hence, flow rates at the mitral valve and aorta during the systole and diastole phase are assumed to be zero. However, in the simulation, there is outflow during the systole for the PV.

### Analysis of vortex dynamics

**Figure 7:**
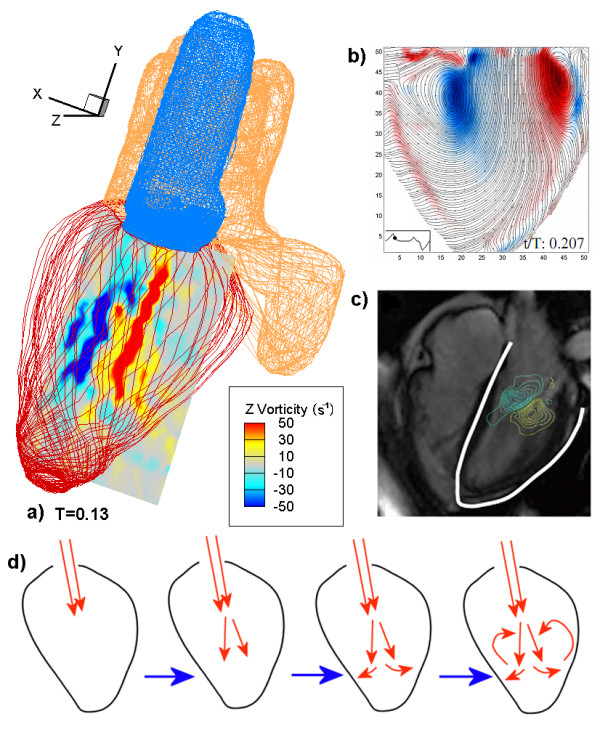
**Visualization of the vortices in the LV through different approaches**. a) Vorticity contours at z = 0.56 (along the same plane as PV) and T = 0.13 using the CMS. b) Experimental results of vorticity fields from Fortini et al. [8]. c) Vorticity contours from the current 4-D PC-MRI system. d) Simplified evolution of flow through a single axisymmetric opening valve.

The vortex dynamics is the most important characteristics in LV. Figure 7a shows the 2-D vorticity of CMS at T = 0.13, near the early diastolic phase. The 2-D vorticity fields (Z vorticity) are obtained by extracting a slice of the heart along the z direction using:(6)

Defining the two edges of the heart as *z *= 0 and 1, the cross-section of the vorticity contour comes from the location *z *= 0.56 (Figure 7a). This slice is chosen because it is on the same plane as the PV and clearly shows the vorticity variation. Negative and positive vortices are shown as blue and red, respectively. The negative vortex is generated on the left while the positive one is on the right. Further comparison with the experimental and clinical data is presented in Figure 7b-c, which show the experimental results of vorticity fields from Fortini et al. [8] and the vorticity contours from the current 4-D PC-MRI system.

### Kinetic energy

**Figure 8:**
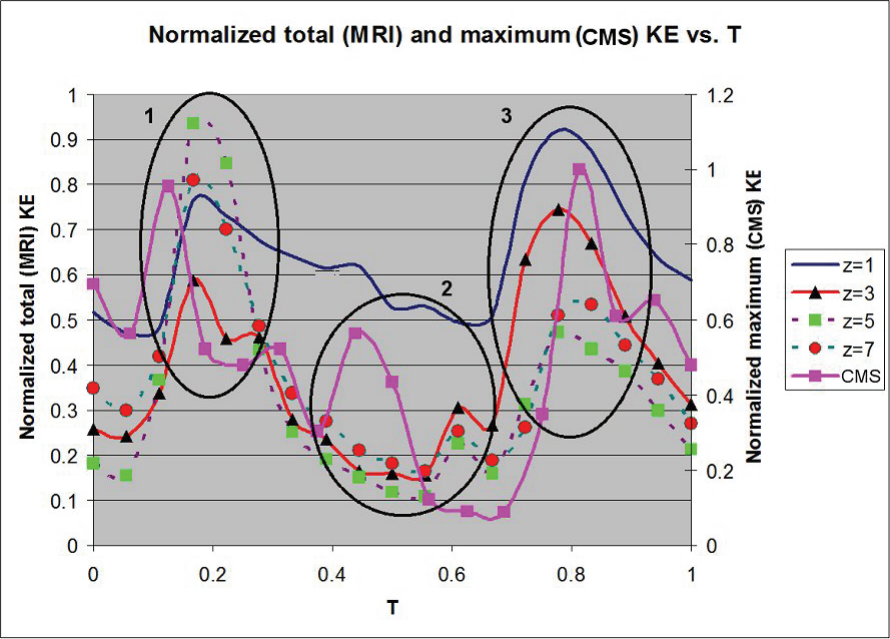
**The comparison of Kinetic Energy (KE) between the 4-D PC-MRI system and CMS**. Normalized total KE at different slices and maximum KE at slice z = 0.56, obtained from the 4-D PC-MRI system and CMS, respectively.

**Figure 9:**
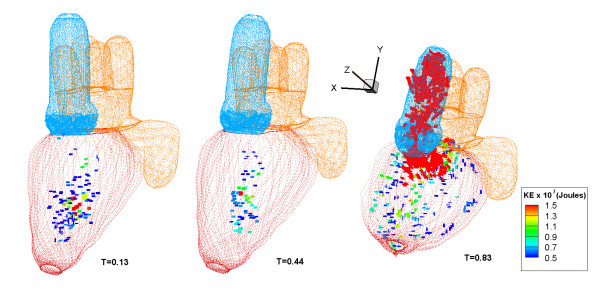
**The KE of the markers in the LV for CMS at different times showing the three peaks**. KE values below 3.0 × 10^-7 ^joules are not shown. The color indicates the level of its KE.

The variation of kinetic energy (KE) of blood flow in the LV indicates the work of the pumping heart. Figure 8 shows the normalized maximum KE, represented by the magnitude of, from the CMS simulation and the total KE from the 4-D PC-MRI data for one cardiac cycle in the LV. A particular plane at *z *= 0.56 is selected in the CMS simulation because it clearly captures the variation of the KE. The figure shows three peaks of the maximum KE at T = 0.13, 0.44 and 0.83 (three black circles 1-3). For the comparison, four different sections out of eight from the 4-D PC-MRI are also chosen to calculate the total KE. Figure 9 shows the actual locations where the maximum KE occurs, which corresponds to the vorticity and energy region. The markers show three distinct peaks. The KE values below 3.0 × 10^-7 ^joules are not shown. We also use colors to indicate the KE level.

## Discussion

### Hemodynamic comparison

We discussed the flow rates through the PV and aorta in the simulation (Figure 5). The large magnitude shown by the red circle 3 (-50 cm^3^/s) during the S-wave seems plausible. The magnitude should be similar to that of the earlier outflow (around T = 0.45) because the mitral valve is closed at this time, as shown in the bottom left of Figure 5 (small green circle). Hence, there is no additional blood travelling through the mitral valve to the LA and out to the PV. In an actual heart, the mitral valve opens to allow blood to flow into the LV during diastole, as shown by the green circle at the top right hand side of Figure 5. Under normal conditions, the mitral valve should prevent backflow during the systolic phase. Although the mitral valve seems to be nearly closed, the 3-D heart model is unable to prevent backflow during the systole phase (also known as mitral valve dysfunction). It is natural that the modelled heart cannot form a fully closed value, which is constructed based on points, fibres, groups and brunches (unlike the usual structured or unstructured grid model). The opening and closing of the valve is due to FSI. The resultant force on the valve may not be sufficient to exactly close it unless a specific constraint is imposed. Lastly, Kovacs [21] mentions that the layout of the fibres and fibre points may need to be more refined in some regions than others to prevent leakage. This is due to the stretching of the regions. Further analysis of the similarity to corresponding heart problems is helpful. In some diseased conditions such as LV dilatation, mitral valve dysfunction, or pulmonary arterial hypertension, large in/out flow variations are observed clinically, consistent with these idealized constant RPBC.

The comparison in Figure 6 shows that the magnitude of PV flow rate is generally twice as high as that of Fortini et al. [8]. The variation is not as pronounced in the PV flow rate between T = 0 and 0.3; it decreases slowly because of the prolonged filling phase to ensure a balanced momentum. For the aorta, the comparison with flow data from Fortini et al. [8] shows similar outflow during the systolic phase. The high outflow at the aorta occurs at a similar time (T = 0.82), indicating that the CMS simulates various phases of the cardiac cycle. However, the aorta flow rate is much higher than that of Fortini et al. [8] (-5.0 compared to -1.0 in Figure 6). The inflow during diastole in their experiment is similar to the outflow during systole. The same occurs in the CMS simulation, although its magnitude is much larger and will most likely conserve the prolonged filled mass flow rate numerically. Further investigation is still required to verify this speculation.

### Analysis of vortex dynamic

This section discusses the evolution of vorticity in the LV throughout the entire cardiac cycle and emphasizes vortex dynamics in the LV of the heart during diastole. As mentioned by Domenichini et al. [9], many researches [15,16] confirm that the vortices generated in the LV are significant in heart functionality. Pierrakos and Vlachos [26] discussed vortex formation during the diastole period and showed that fluid transport is more efficient by vortex ring formation, compared to a steady, straight jet of fluid.

The formation of the vortices and their respective locations can be explained using Figure 7d. The blood flows into the LV through the mitral valve opening. As the blood enters the LV, the larger surrounding space allows it to "spread" after traveling a short distance. Because the LV is an enclosed space, the blood interacts with the wall and is constrained to "roll back" giving the vortices shown. In this simplified illustration of an axisymmetric valve, the valve in the modeled heart is non-axisymmetric. Numerous experiments also show the pair of vortices. Fortini et al. [8] modeled the LV using a conical sack made of silicone rubber. Their results showed two vortices of opposite signs, similar to the simulation. The MRI image obtained from the 4-D PC-MRI system also shows the pair of vortices. Our simulation results tally well with the two experimental results (Figure 7b and 7c). However, Fortini et al. [8] also argued that, as the diastolic phase proceeds, the left negatively signed vortex (blue) would grow stronger than the right positively signed one. They suspected that the right (red) vortex interacts viscously with the wall of the LV, slows down, and diminishes in size. The same process does not result in the left (blue) vortex and hence it is able to grow larger. Unfortunately, this is not evident in the current simulation. We suspect the current simulation may not be able to accurately simulate the viscous interaction of the vortex with the wall boundary, which may require a finer mesh.

### Kinetic energy

Figure 8 shows a large volume of blood flow into the LV through the PV in the first two timings (T = 0.13 and 0.44). This generates vortices which help to minimize energy dissipation during flow transport [27]. The first peak corresponds to a high blood inflow into the LV during the initial diastole (also known as E-wave) while the second lower peak (A-wave) corresponds to atrial contraction. Because a higher flow rate is akin to higher kinetic energy, one can also compare with the time varying flow rate (Figure 6). These two peaks are also observed in the data of Fortini et al [8]. In this case, the peaks (E and A) occurred at approximately T = 0.16 and 0.62 (interval of 0.46 T). Hence, its time interval is also slightly longer. This may result from a number of possibilities. The clinical data may not be universal because of variations in patients, timing, and other factors. From the simulation, the imposed boundary conditions influence the variation in KE as well. The third peak in Figure 8 corresponds to the large outflow of oxygenated blood through the aorta valve during the systolic period. This is also captured in Figure 6, which is approximately T = 0.83.

The normalized total KE of the heart using the 4-D PC-MRI system is also compared in Figure 8. Due to the limitation of the system, it is not possible to obtain maximum KE only in the LV. Hence, a direct comparison between the 4-D PC-MRI and CMS results is impractical in Figure 8. Nevertheless, the qualitative information can be extracted from the total KE based on a variety of horizontal sections. The z = 5 plane of the 4-D PC-MRI image (the slice at approximately the center of the heart in the z-plane) is similar in position to the plane shown by the CMS result in Figure 8 (z = 0.56). There are also three peaks, with the second peak being less obvious (T = 0.65) in the 4-D PC-MRI graph. These correspond to the initial diastolic, atrial contraction, and systolic phases, demonstrating that the CMS is able to show different peaks reasonably. The timings for the first and third peaks are similar between the two results. However, the second peak occurs at an earlier time of T = 0.44 for the CMS result. More investigations are required due to several uncertainties in either data or simulation (e.g. various categories of volunteers such as male and female, age, and the boundary conditions imposed).

## Conclusions

The current study implemented the CMS based on the code by Mcqueen and Peskin [14]. We compared and evaluated the results in terms of hemodynamic, vorticity visualization, and kinetic energy analysis. The simulation properly captures the peaks during the E-, A-, and S-waves. However, the actual magnitudes do not tally well. Outflow is detected at the PV during the filling phase. This occurs despite the closure of the mitral valve. During systole, outflow occurs at the aorta due to oxygenated blood rushing out to different parts of the body.

Modeled vorticity fields show similar results compared to the experiments conducted by Fortini et al. [8]. Both simulation and experimental results show two oppositely signed vortices in the LV. However, the simulation does not capture the difference in size of the left and right vortices. In terms of kinetic energy, the simulation results show a similar number of peaks (three) as those found in the volunteer from the 4D PC-MRI system. The result also correctly reflects the relative heights of the first two peaks (first higher than the second) compared with the clinical data. Some differences in the timing of the peaks still exist, although it is still uncertain due to the variation in patients.

The results show that using a simple RPBC is able to capture some of the essential variations found in the clinical data in some basic analysis, which encourages further aid of physician's diagnostics using the CMS approach. These include the peaks captured in Figure 6/and8, and the vortex pair in Figure 7. Some discrepancies between the simulation and clinical data are still present, such as the occurrence of backflow even when the mitral valve is closed, difference in the magnitude of aorta flow rate (Figure 6), and the timing difference in the second peak during the comparison of the KE. Further detailed investigations are required to determine the actual causes behind the discrepancies. More sensitivity tests should be performed further in terms of the RPBC, boundary conditions and resolution dependence of CMS and the characteristics of vortex structure from the 4D PC-MRI system in order to complete the realistic application of the integrated system.

The presented approach establishes a first-step framework of a practical patient-specific CMS, which comprises a 3-D CFD model to simulate the heart and the 4-D PC-MRI system. At this stage, the 4-D PC-MRI system is used for verification purpose rather than input. This study brings us closer to our goal of developing a practical patient-specific CMS, which will be pursued next. In practice, conducting a patient-specific CMS in advance can accurately predict and visualize the blood flow in the heart, thus providing a general understanding and adequate evaluation of the heart flow dynamics noninvasively. Since the 3-D modelling component of this patient-specific CMS is still computationally expensive, this system can be used ideally for the purpose of pre-operation evaluation or surgical planning. It can solve the common echocardiographic windows problem for many patients. Most important of all, the vortex dynamic and other complicated flow dynamics can be comprehensively revealed and provided to the physicians for necessary treatment. This can greatly simplify the diagnostic process compared to using MRI, echocardiography or other techniques, which have difficulties as mentioned earlier.

## Consent

Written informed consent was obtained from the patient for publication of this study and accompanying images. A copy of the written consent is available for review by the Editor-in-Chief of this journal written informed consent was obtained from the patient for publication of this report.

## Competing interests

The authors declare that they have no competing interests.

## Authors' contributions

WBT and YHT conceived the study, participated in the design of the study and drafted the manuscript. LYL and WYT provided the 4-D MRI data and helped to explain the blood circulation in the heart. WBT and YHT carried out the numerical simulations and participated in the design and coordination of the work and helped to revise critically the manuscript. All authors read and approved the final manuscript.
